# A Compound Computational Model for Filling-In Processes Triggered by Edges: Watercolor Illusions

**DOI:** 10.3389/fnins.2019.00225

**Published:** 2019-03-22

**Authors:** Hadar Cohen-Duwek, Hedva Spitzer

**Affiliations:** Vision Research Laboratory, School of Electrical Engineering, Tel-Aviv University, Tel Aviv, Israel

**Keywords:** computational models, watercolor effect, filling-in, diffusion process, visual system mechanism

## Abstract

The goal of our research was to develop a compound computational model with the ability to predict different variations of the “watercolor effects” and additional filling-in effects that are triggered by edges. The model is based on a filling-in mechanism solved by a Poisson equation, which considers the different gradients as “heat sources” after the gradients modification. The biased (modified) contours (edges) are ranked and determined according to their dominancy across the different chromatic and achromatic channels. The color and intensity of the perceived surface are calculated through a diffusive filling-in process of color triggered by the enhanced and biased edges of stimulus formed as a result of oriented double-opponent receptive fields. The model can successfully predict both the assimilative and non-assimilative watercolor effects, as well as a number of “conflicting” visual effects. Furthermore, the model can also predict the classic Craik–O'Brien–Cornsweet (COC) effect. In summary, our proposed computational model is able to predict most of the “conflicting” filling-in effects that derive from edges that have been recently described in the literature, and thus supports the theory that a shared visual mechanism is responsible for the vast variety of the “conflicting” filling-in effects that derive from edges.

## Introduction

One of the most important goals of the higher levels of visual system processing is to reconstruct an appropriate representation of a surface after edge detection is performed by early vision. Such tasks are attributed to the opponent receptive fields in the retina and in the lateral geniculate nucleus (LGN). The visual system processing involves the cortical double-opponent as well as the simple and complex receptive fields, which perform non-oriented and oriented edge detection of both chromatic and non-chromatic edges (von der Heydt et al., 2003).

There are a number of visual phenomena and illusions that can provide information about the mechanisms that enable the reconstruction of surfaces from their edges. These include the watercolor illusions (Pinna et al., 2001) and the Craik-O'Brien-Cornsweet illusion (Cornsweet, 1970). In this study we will concentrate mainly on developing a computational model for the watercolor illusions to include a prediction of “conflicting” watercolor effects.

The Watercolor Effect described in the literature refers to a phenomenon involving assimilative color spreading into an achromatic area, produced by a pair of heterochromatic contours surrounding an achromatic surface area (Pinna et al., 2001; Pinna, 2008; Devinck and Spillmann, 2009). The coloration extends up to about 45° (visual degree) and is approximately uniform (Pinna et al., 2001).

There have been many studies that investigated the chromatic and the luminance parameters required for the two inducing contours and for the inducing contours and background of the watercolor effect (Pinna et al., 2001; Devinck et al., 2005, 2006, 2014; Pinna and Grossberg, 2005; Pinna and Reeves, 2006; Tanca et al., 2010; Cao et al., 2011; Devinck and Knoblauch, 2012; Hazenberg and van Lier, 2013; Coia and Crognale, 2014; Coia et al., 2014). The conclusion was that even though many color combinations can produce the effect, the strongest result is induced by a combination of complementary colors. The studies of Pinna et al. (2001), Devinck et al. (2005, 2006) characterized these findings as assimilation effects (i.e., the perceived color is similar to the color of the nearest inducer). Reversing the colors of the two inducing contours, reverses the resulting perceived colors accordingly (Pinna, 2008).

However, a non-assimilation effect of coloration has also been discussed (Pinna, 2006; Kitaoka, 2007). Pinna (2006) reported that if one of the inducers is achromatic, while the other is chromatic, the induced color can be complementary to that of the chromatic inducer. Kitaoka (2007) demonstrated that a combination of red-magenta or green-cyan can give rise to a yellowish coloration, indicating that the perceived effect may not be completely attributable to assimilation effects. Indeed, an achromatic watercolor effect has been recently proved to exist, albeit with a lower magnitude than the chromatic watercolor effect (Cao et al., 2011).

The only computational model that has been reported to explain the watercolor effect is called the “Form And Color And Depth” (FACADE) model (Grossberg and Mingolla, 1985) and is based on neurophysiological evidence from neurons in the cortical areas V1–V4 (Pinna and Grossberg, 2005). This model also attempts to explain a number of other visual phenomena including the Kaniza illusion (Kanizsa, 1976), neon color spreading (van Tuijl and Leeuwenberg, 1979), simultaneous contrast, and assimilation effects. FACADE describes two main visual processing systems: a boundary contour system (BCS) that processes boundary or edge information; and a feature contour system (FCS) that uses information from the BCS to control the spreading (filling-in) of surface properties such as color and brightness. According to this model, higher contrast boundaries in the BCS inhibit lower-contrast boundaries thereby enabling color to flow out through weaker boundaries.

A number of studies have proposed the FACADE model as a possible mechanism for predicting the watercolor effect since it explains some of the properties of the phenomenon (Grossberg et al., 2005; Pinna and Grossberg, 2005; Pinna, 2006; Tanca et al., 2010). However, neither the mathematical equations of the FACADE model nor other previous studies have succeeded in simulating and predicting all the experimental findings concerning the watercolor effect. Moreover, the FACADE model cannot predict the non-assimilative version of the watercolor effect (Pinna et al., 2001; Kitaoka, 2007; Hazenberg and van Lier, 2013; Kimura and Kuroki, 2014a). Kitaoka (2007) observed that in the non-assimilative watercolor effect, the induced color becomes more prominent when the outer contour has a higher luminance (and thus a lower-contrast with respect to the white background) than the inner contour. In this case, the BCS in the FACADE model would be expected to inhibit the boundaries of the lower-contrast outer contour and permit the color of the outer contour to spread out. This prediction is not supported by the actual perceived color as demonstrated in **Figure 5**, where a yellowish color spreads in and there is no perceived magenta color that spreads out, as the FACADE model would predict.

At present, the visual mechanisms responsible for the watercolor effect are still unknown and the watercolor effect “presents a significant challenge to any complete model of chromatic assimilation” (Devinck et al., 2014).

In their study on the watercolor effect, Knoblauch et al. (Devinck et al., 2014) summarized the requirements for a future computational model: “In a hierarchical model, two other steps need to be considered, surface detection then color filling-in.”

In this study, we present a computational model, which detects edges through biological receptive fields, modifies them, and then applies them as a trigger for a diffusive filling-in process. The objective of the model is to predict both the assimilative and the non-assimilative configurations of the watercolor effect.

## Computational Model

**Figure 1:**
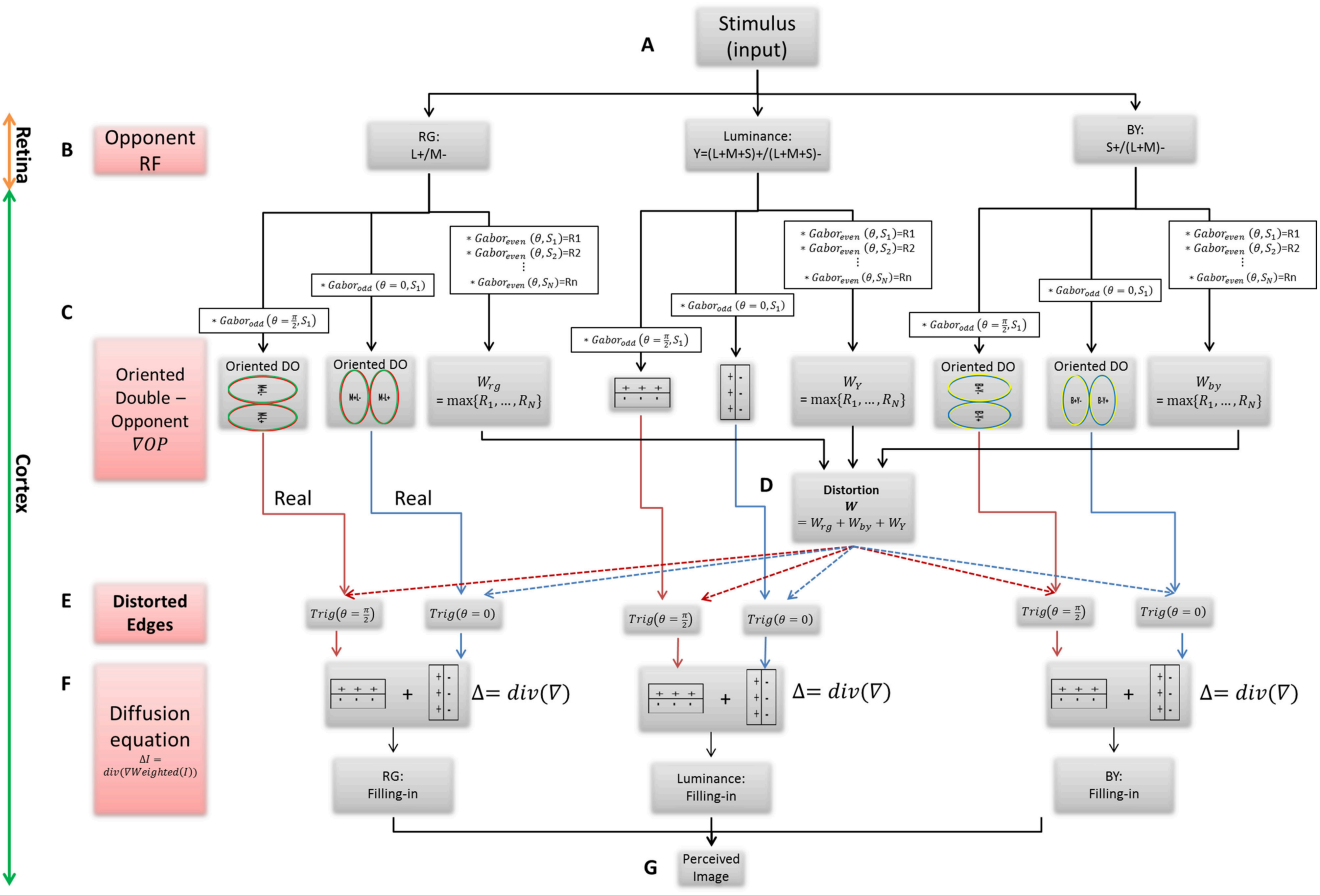
The Flowchart of the suggested filling-in model. **(A)** The chromatic stimulus. **(B)** The opponent RFs, which are used as the first derivative in the chromatic and chromatic channels. **(C)** The oriented RFs, which represent the real chromatic gradients of the stimulus. **(D)** The calculation of weight function W for the modification of the gradients. **(E)** The calculation of the triggers for the filling-in process, i.e., the real and the modified edges where each of them exists in the x and y directions. **(F)** The filling-in process, calculated by the Poisson equation. **(G)** The solution of the diffusion equation yields to the perceived image.

The main building blocks of the model are: (A) The inducing stimulus (B) The chromatic and achromatic opponent receptive fields (RFs). (C) The oriented double-opponent RFs, which detect chromatic and achromatic edges. (D) Calculation of the modification value through determination of the dominant chromatic/achromatic stimulus edge among several edges, which have different spatial scales. (E) Calculation of the new modified edges that trigger a diffusive filling-in process. (F) The filling-in process, performed by solving the Poisson equation. (G) The perceived afterimage of both the assimilative and the non-assimilative watercolor effects (Figures 1A-G).

### Model Assumptions

The model is based on the following assumptions: (A) The visual system needs to reconstruct surfaces that are not represented in the early vision stages, which perform chromatic and achromatic edge detection (in the retina and the cortical V1 and V2 areas). In addition, we assume that in cases such as the watercolor stimuli, the visual system performs filling-in processes in order to make an “educated guess” and to reconstruct surfaces. (B) Each edge triggers a diffusion process and determines its color (Cohen-Duwek and Spitzer, 2018). (C) The trigger for the diffusion process is determined by the interactions between the gradients of the image, i.e., the gradients between the inner contour (IC) and the outer contour (OC), the gradients between the IC and the background, and between the OC and the background. The exact contribution of each gradient is determined automatically according to the chromatic and achromatic stimulus. (D) The visual system uses separated chromatic opponent channels [L/M, (L+M)/S and achromatic], in order to process each contrast color pathway separately (Kandel et al., 2012). This assumption is in agreement with experimental studies which claimed that the (L/M) and S-cones are regulated differently with respect to the watercolor effect (Devinck et al., 2005; Kimura and Kuroki, 2014a,b). (E) The chromatic channels are mediated by the Luminance channel (the achromatic channel). This assumption is supported by the observation that there is color spreading in response to a stimulus where both the IC and OC have the same color (hue) but a different luminance (Devinck et al., 2006).

### Rationale for the Model

The early stages of the visual system, the retina, and the early visual areas V1 and V2, have receptive fields (RFs) that mainly detect edges. In the retina, for example, the opponent receptive fields perform a Difference of Gaussian (DOG) operation, which is approximately a second spatial derivative while the chromatic retinal opponent RFs performs derivatives on the color domain. The simple and complex RFs in the V1 and V2 areas perform oriented edge detection. It has been assumed that at higher visual processing levels, the system acts to reconstruct the surfaces that are not represented (lacked) by the early visual areas. In order to perceive the physical world and not only its edges/gradients, the system (visual system) needs to reconstruct the image from its edges (von der Heydt et al., 2003). To mimic the original surfaces, the system could use the image's original gradients (in a similar fashion to that used in the engineering world, i.e., by solving the Poisson equation or by any parallel method (Bertalmio et al., 2000; Pérez et al., 2003). However, we now believe that in addition, the visual system also performs additional tasks, which can be regarded as “educated guesses” in order to enhance important information in the scene. Examples of such “educated guesses” include: edge completion, detection of occluded objects in the image, and the interpretation of specific gradients as indicative of adjacent surfaces. The watercolor stimulus is such an example of specific edges, where the visual system supplies a guess regarding the chromatic surface. We suggest here, that this educated guess calculation is achieved by modifying the gradients and modifying the weights of the image gradients. In addition, we describe a set of rules that determine how the weights are calculated in the context of the stimulus.

In order to produce the chromatic (or the achromatic) diffusion process, the visual system needs to enhance or change the original gradients in order to obtain an image which creates the perception and avoids a return to the original image. Based on psychophysical findings, the model assumes that the chromatic edges, which determine the filling-in effect, are significantly influenced by the intensity and by the chromaticity of the contours (IC and OC) (Pinna et al., 2001; Devinck et al., 2005, 2006; Pinna and Grossberg, 2005; Pinna and Reeves, 2006; Cao et al., 2011; Hazenberg and van Lier, 2013; Coia and Crognale, 2014; Kimura and Kuroki, 2014a,b).

### The Watercolor Stimulus

The input of the model comprises the watercolor stimulus and its variations, which are composed of a pair of heterochromatic contours surrounding achromatic surface areas, Figure 1A.

### Chromatic and Achromatic Opponent RF

The first component of the model (Figure 1B) is designed to simulate the opponent receptive fields (Nicholls et al., 2001). The spatial response profile of the retinal ganglion RF is expressed by the commonly used DOG. The “center” signals for the three spectral regions, *L*, *M*, and *S*, (Long, Medium, and Short wavelength sensitivity, respectively) that feed the retinal ganglion cells, are defined as the integral of the cone quantum catches, *L*_*cone*_, *M*_*cone*_, *and S*_*cone*_ with a Gaussian decaying spatial weight function (Shapley and Enroth-Cugell, 1984; Spitzer and Barkan, 2005):

(1) $$\begin{array}{l} i_{c}=i_{cone}*f_{c};\;i\;\epsilon \;\left\{L,M,S\right\}\\ i_{s}=i_{cone}*f_{s};\;i\;\epsilon \;\left\{L,M,S\right\}\\ f_{j}=\frac{\text{exp}\left(\frac{-\left(x^{2}+y^{2}\right)}{\rho_{j}^{2}}\right)}{\pi \rho_{j}^{2}},\;j\in \left\{c,s\right\} \end{array}$$

Where *L*_*c*_, *M*_*c*_ *and S*_*c*_ represent the response of the center area of the receptive field of each cell type, Equation 1. *L*_*s*_, *M*_*s*_, *and S*_*s*_ represent the surround sub-region of these receptive fields. ρ_*c*_ and ρ_*s*_ represents the radius of the center and the surround regions, of the receptive field of the color-coding cells, respectively. *f*_*c*_ and *f*_*s*_ are the center and surround Gaussian profiles, respectively and ^*^ represents the convolution operation.

For the center-surround cells, the opponent responses are expressed as: $OP_{L^{+}M^{-}}$, $OP_{S^{+}{\left(L+M\right)}^{-}}$ and *Y* (for the summation of the L, M, and S channels) in order to express the Luminance channel.

(2) $$\begin{array}{l} OP_{RG}:\;OP_{L^{+}M^{-}}=L_{c}-M_{s}\;(\text{Red}-\text{Green channel) }\\ OP_{BY}:\;OP_{S^{+}{\left(L+M\right)}^{-}}=S_{c}-{\left(L+M\right)}_{s}(\text{Blue}-\text{YellowChannel) }\\ Y={L_{c}+M_{c}+S}_{c}\;\left(\text{Luminance channel}\right) \end{array}$$

Where *L*_*c*_, *M*_*c*_, *s*_*c*_, *L*_*s*_, *M*_*s*_, and *S*_*c*_ are the cell responses to the receptive filled sub-regions: center and surround, Equation (1).

### Oriented Double-Opponent RF

The color coding of the opponent receptive fields, Equation (2), encodes color contrast, but not spatial contrast. In other words, the color opponent receptive fields are able to differentiate between colors, but cannot detect spatial gradients or edges (Conway, 2001; Spitzer and Barkan, 2005; Conway and Livingstone, 2006; Conway et al., 2010). The double opponent receptive fields, however, are sensitive to both spatial and chromatic gradients (Spitzer and Barkan, 2005) since they have color opponent receptive fields both at the center and in the surround RF regions (Shapley and Hawken, 2011). A large number of studies have reported that many double-opponent neurons are also orientation-selective (Thorell et al., 1984; Conway, 2001; Johnson et al., 2001, 2008; Horwitz et al., 2007; Solomon and Lennie, 2007; Conway et al., 2010). Accordingly, the model takes into account the oriented double opponent RF, ODO, to the three opponent RF channels, $OP_{L^{+}M^{-}}$, $OP_{S^{+}{\left(L+M\right)}^{-}}$, and Y (Conway and Livingstone, 2006), Equation (2). We modeled this chromatic RF structure, $ODO_{L^{+}M^{-}}$, $ODO_{S^{+}{\left(L+M\right)}^{-}}$ and *OY* by a convolution between the Gabor function and the opponent responses, Equation (3), Figure 1C. It should be noted that previous work indicates that by using the linear Gabor function, we neglect some non-linearities e.g., half wave rectification in the simple cells and full rectification in the complex cells, in the neuronal responses (Movshon et al., 1978; Spitzer and Hochstein, 1985).

(3) $$\begin{array}{l} \;ODO_{L^{+}M^{-}}=OP_{L^{+}M^{-}}*Gabor_{odd,\theta ,\sigma }\\ ODO_{S^{+}{\left(L+M\right)}^{-}}=OP_{S^{+}{\left(L+M\right)}^{-}}*Gabor_{odd,\theta ,\sigma }\\ \;OY=Y*Gabor_{odd,\theta ,\sigma } \end{array}$$

(4) $$\begin{array}{l} \;Gabor_{odd,\theta ,\sigma }=\text{exp}\left(\frac{-\left(x^{\prime 2}+y^{\prime 2}\right)}{2\sigma^{2}}\right)\text{sin}\left(2\pi {\;x}^{\prime }\right)\\ Gabor_{even,\theta ,\sigma }=\text{exp}\left(\frac{-\left(x^{\prime 2}+y^{\prime 2}\right)}{2\sigma^{2}}\right)\text{cos}\left(2\pi \;x^{\prime }\right)\\ \text{Where}:\;x^{\prime }=xcos\left(\theta \right)+ysin\left(\theta \right)\\ \;y^{\prime }=-xsin\left(\theta \right)+ycos\left(\theta \right) \end{array}$$

This opponency in both spatial and chromatic properties produces a spatio-oriented-chromatic edge detector, Equation (3).

Where θ represents the orientation of the normal to the parallel stripes of a Gabor function and σ is the standard deviation of the Gaussian envelope of the Gabor function.

### Gradient Weights

We chose to express this property of gradient modification by adding weighted functions to the Oriented-double-opponent RF (Figure 1D). The model modifies the original gradients (Equation 3) by multiplying the double-opponent responses by the weight function, Equation (6), Figure 1D. In order to calculate the weight functions, several Gabor-filters on different scales [different standard deviations, σ, Equation (5)] are calculated and the maximum response to a specific Gabor RF scale is chosen as the weight function for each channel separately, Equation (6). This maximum response represents the dominant gradient in the image, which is used by the model to determine the strongest effect on the diffusion process. This determination of the strongest effect (i.e., the strongest edge in the stimulus) is in agreement with previously reported psychophysical findings (Pinna et al., 2001; Devinck et al., 2005, 2006; Kimura and Kuroki, 2014a,b). The multiplication operation of the chosen weight is done with a 2D Gabor filter, Equation (5). (It should be noted that we could also obtain good results by making a summation of the responses from all scales).

(5) $$\begin{array}{l} \;R_{RG,i}=|OP_{RG}*Gabor_{even}\left(\theta ,\sigma_{i}\right)|\\ \;R_{BY,i}=|OP_{BY}*Gabor_{even}\left(\theta ,\sigma_{i}\right)\;|\\ \;R_{Luminance,i}=|Y*Gabor_{even}\left(\theta ,\sigma_{i}\right)| \end{array}$$

Where σ_*i*_ represents different standard deviations of the Gaussian envelope (different scales).

(6) $$\begin{array}{l} W_{RG}\left(i,j\right)=\max\left\{R_{RG,1}\left(i,j\right),R_{RG,2}\left(i,j\right),\ldots ,R_{RG,N}\left(i,j\right)\right\}\\ W_{BY}\left(i,j\right)=\max\left\{R_{BY,1}\left(i,j\right),R_{BY,2}\left(i,j\right),\ldots ,R_{BY,N}\left(i,j\right)\right\}\\ \;W_{Y}(i,j)=\max\{R_{\text{Luminance},1}(i,j),R_{\text{Luminance},2}(i,j),\ldots ,\\ R_{\text{Luminance},N}(i,j)\} \end{array}$$

Where *W*_*RG*_, *W*_*BY*_, and *W*_*Lum*_ are the maximal responses among the several scales at each channel.

This calculation is done separately for both the chromatic channels and the achromatic channels (RG, BY, and Y). After determining which scale yields the strongest response at each channel, the three responses are summarized across the channels, Equation (7), to reflect a combination of all the edges in each spatial location. In other words, the weight function W, for each spatial location in the image (or stimulus), is taken as the normalized sum of the maxima, values from the strongest response scale, across all the channels, Equation (7).

(7) $$\begin{array}{l} W=W_{RG}+W_{BY}+W_{Y} \end{array}$$

This calculation procedure can detect the middle chromatic (or achromatic) edge between the two contours (IC and OC), which are the triggers for the diffusion process. This detection is possible because in most cases, the dominant edge is a coarse edge, which contains the edge that is adjacent to the inner and the outer region. The center of this coarse region is often the edge between the two chromatic contours in the watercolor stimuli.

### The Diffusion Triggers (Second Derivative)

The trigger for the diffusion process consists of the sum of two components: the modification component (β) and the “real” (α) oriented double-opponent RF component, Equation (8). These modification components are added separately for each orientation directions and then, the modified gradients are convolved again with an odd Gabor filter (in the same orientation, θ), Equation (10), in order to perform a second derivative. Both derivative direction (x and y axis, θ = 0 and $\theta =\frac{\pi }{2})$ are then summarized in order to create the divergence, Equation (10), Figure 1F, which is then used as the trigger for the diffusion process in all the required directions, Equation (10), across each of the channels. The trigger for the diffusion process is the oriented-double-opponent response, Equation (3), multiplied by the weight function (W) in each individual channel, Figure 1E, Equation (8).

(8) $$\begin{array}{l} Trig_{RG}=ODO_{RG}\cdot \left(\alpha +\text{β}W\left(x,y\right)\right)\\ Trig_{BY}=ODO_{BY}\cdot \left(\alpha +\text{β}W\right)\\ \;Trig_{Y}=OY\cdot \left(\alpha +\text{β}W\right) \end{array}$$

Where α and β are constants and α > β. *Trig*_*RG*_, *Trig*_*BY*_, and *Trig*_*Y*_ are the diffusion triggers in each channel.

Note that the results of the above equations change only the weights of the ODO (Equation 3) responses, and therefore their spatial properties and polarities are retained. According to the suggested model, the prominent gradient makes the strongest contribution to the filling-in process, Equation (7). However, the other two gradients also contribute to the filling-in process, due to the chromatic and achromatic strength of their gradients. This consideration of the different gradients is in agreement with the Weber contrast rule (Kimura and Kuroki, 2014a).

### Filling-In Process

The filling-in process is expressed by the diffusion (or heat) Equation (10) (Weickert, 1998), and is determined according to the weighted triggers, Equation (8), Figure 1E. The model assumes that the filling-in process represents “isomorphic diffusion” (von der Heydt et al., 2003; Cohen-Duwek and Spitzer, 2018), although it does not necessarily negate other possible filling-in mechanisms, such as “edge integration” (Rudd, 2014). This filling-in process is reminiscent of the physical diffusion process, where the signals spread in all directions, until “blocked” by another heat source (image edges). We would like to emphasize that this type of filling-in infers that the borders (chromatic or achromatic) do not function primarily as blockers, but instead they act as heat sources that can trigger the diffusion. We would like to emphasize that this type of filling-in infers that the borders (chromatic or achromatic) do not function primarily as blockers, but instead they act as heat sources that can trigger the diffusion, and then spread in opposite directions and thus trap the diffused color. The diffusion spread, therefore, will be blocked by the heat source, in such a case. These principles are applied in our model through the well-known diffusion equation (Weickert, 1998):

(9) $$\begin{array}{l} \frac{\partial \text{I}\left(x,y,t\right)}{\partial t}-D\nabla^{2}\text{I}\left(x,y,t\right)={\text{h}}_{s}=-\text{div}\left(Trig_{c}\right);\\ \text{ where c}=\left\{L^{+}M^{-},S^{+}{\left(L+M\right)}^{-},\text{Y}\right\} \end{array}$$

where I(*x, y, t*) denotes the image in a space-time location (*x, y, t*), D is the diffusion (or heat) coefficient, and h_*s*_ represents a heat source. The time course of the perceived image is assumed to be very fast, in accordance with previous reports (Pinna et al., 2001). This time course is also termed “immediate filling-in” (von der Heydt et al., 2003).

Following this assumption, for the sake of simplicity, we can ignore the fast-dynamic stages of the diffusion equation, and therefore compute only the steady-state stage of the diffusion process. Consequently, the diffusion (heat) Equation (5) is reduced to the Poisson Equation (10).

(10) $$D\nabla^{2}\text{I}=-{\text{h}}_{s}=\text{div}\left(Trig_{c}\right);\text{ where c}=\{\text{RG},\text{BY},\text{Y}\}$$

(11) $$\begin{array}{l} D\nabla^{2}\text{I}=\text{div}\left(\left(\alpha +\beta W\right)\cdot \text{ODO}\right) \end{array}$$

**Figure 2:**
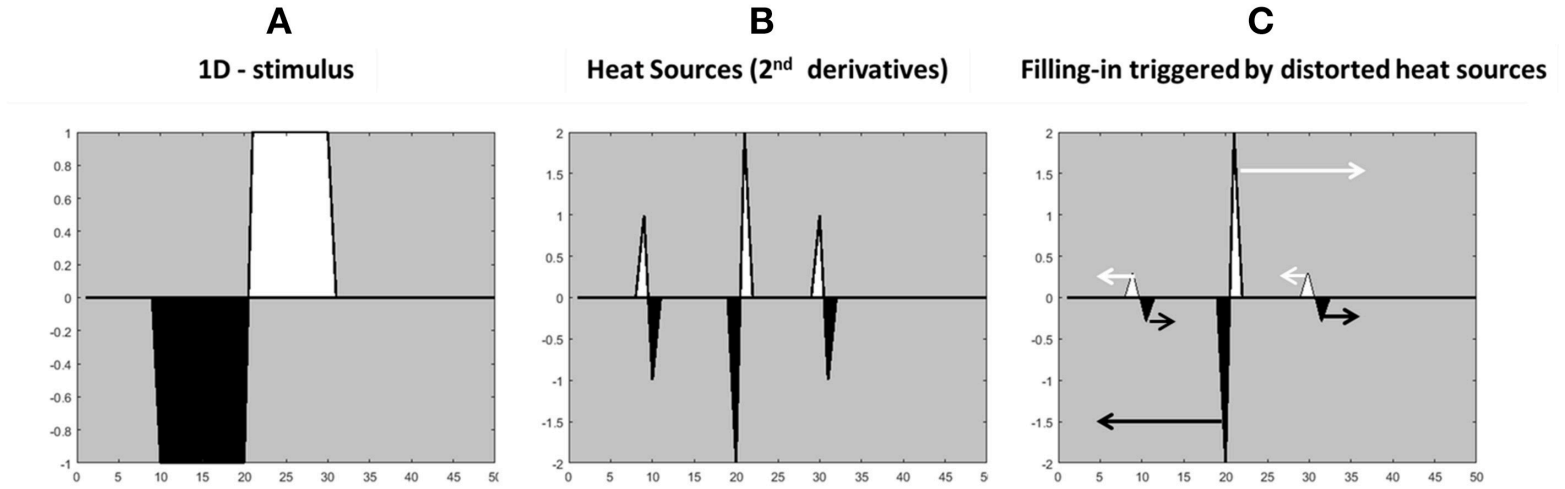
Illustration of the calculation of the edges for the “heat sources” filling-in process from the stimulus gradients. **(A)** 1-D achromatic stimulus with white and black contours. **(B)** The second derivative of the stimulus **(A)**, with a negative sign. **(C)** The modified second derivative of the stimulus **(A)**. The arrows indicate the direction and the color of the diffusion process. The higher heat sources (the gradients in the middle) have a greater influence on the filling-in process.

The “heat sources” are the weighted second derivative of an opponent channel; Figure 1E (weighted oriented-double-opponent). The heat equation (diffusion equation) with heat sources requires second derivatives, reflecting the “heat generation rate” which is the second derivatives of a heat source. Because the edges are playing a role as heat sources, the values near the edges do not decay over time. Since the two adjacent edges operate as heat sources with opposite signs, the conclusion is that they are operating with opposite directions, and therefore the diffusion process of one color (one heat source) cannot diffuse to the “other” direction. This approach is not consistent with previous reports that the edges function as borders that prevent the colors from spreading (Cohen and Grossberg, 1984; Grossberg and Mingolla, 1985, 1987; Pinna and Grossberg, 2005). In the suggested model the derivatives trigger a positive diffusion process toward one side of the spatial derivative and a “negative diffusion” process to the other side of the spatial derivative, Figure 2 demonstrates this type of diffusion, which is considered separately for each color channel.

## Methods

In this section we describe each stage of the model's implementation in detail.

### Opponent RF

For the sake of simplicity, we compute the opponent response of the opponent receptive fields as color-opponent only, where each chromatic encoder has the same spatial resolution. This is computed by an opponent color-transformation (van de Sande et al., 2010), Equation (12). This transformation converts each pixel of the image I_0_, in each chromatic channel R,G, and B into opponent color-space, via the transformation matrix O (van de Sande et al., 2010). In order to obtain more perceptual value in the luminance channel, we have slightly modified the transformation matrix O, and use a = 0.2989, b = 0.5870, and c = 0.1140, instead of using $a=b=c=1/\sqrt{3}$ as originally reported (van de Sande et al., 2010). These values are taken from the Y channel in YUV (or YIQ) color space. The Y represents the Luma information: Y = 0.2989R + 0.5870B + 0.1140C. I_OPPONENT_ = OPPONENT{RGB} as follows:

(12) $$\begin{array}{l} I_{OPPONENT}=\left(\begin{array}{c} O_{RG}\\ O_{YB}\\ O_{Y} \end{array}\right)=\left(\begin{array}{ccc} 1/\sqrt{2} & -1/\sqrt{2} & 0\\ 1/\sqrt{6} & 1/\sqrt{6} & -2/\sqrt{6}\\ a & b & c \end{array}\right)\left(\begin{array}{c} R\\ G\\ B \end{array}\right)\; \end{array}$$

Another perceptual option for the opponent transformation matrix is to use the transformation presented by Wandell (1995),

(13) $$\begin{array}{l} \;I_{OPPONENT}=M_{OpponentW}\left\{M_{LMS}\left\{M_{XYZ}\left\{RGB\right\}\right\}\right\}\\ \;M_{XYZ}=\left(\begin{array}{ccc} 0.4124 & 0.3576 & 0.1805\\ 0.2126 & 0.7152 & 0.0722\\ 0.0193 & 0.1192 & 0.9505 \end{array}\right)\\ \;M_{LMS}=\left(\begin{array}{ccc} 0.2430 & 0.8560 & -0.0440\\ -0.3910 & 1.1650 & 0.0870\\ 0.0100 & -0.0080 & 0.5630 \end{array}\right)\\ M_{OpponentW}=\left(\begin{array}{ccc} 1 & 0 & 0\\ -0.59 & 0.80 & -0.12\\ -0.34 & -0.11 & 0.93 \end{array}\right) \end{array}$$

(14) $$\begin{array}{l} I_{OPPONENT}=\left(\begin{array}{c} O_{Y}\\ O_{RG}\\ O_{YB} \end{array}\right)=\left(\begin{array}{ccc} 0.2814 & 0.6938 & 0.0638\\ -0.0971 & 0.1458 & -0.0250\\ -0.0930 & -0.2529 & 0.4665 \end{array}\right)\left(\begin{array}{c} R\\ G\\ B \end{array}\right) \end{array}$$

These matrix values are calculated from the linear conversion of the RGB color space to the XYZ color space, which is then converted to the LMS color space to which we apply the opponent transformation from Wandell (1995), Equation (13).

where O_RG_, O_YB_, and O_Y_, Equations (12–14) are the new channels of the transformed image I_OPPONENT_. R, G, and B are the red, green, and blue channels of the input image I, respectively.

### Oriented Opponent and Double-Opponent RF

The oriented opponent RFs are modulated as convolution between each opponent channel and an odd Gabor function, Equation (4). For the sake of simplicity, we discretized the Gabor function and instead of computing the exact Gabor functions, we used a discrete derivative filter in two directions, vertical (y-axis, θ = 0), and horizontal (x-axis, $\theta =\frac{\pi }{2}$), Equations (15–16) (Gonzalez and Woods, 2002).

(15) $$\begin{array}{l} Gabor_{odd,x}\approx G_{odd,x}=\left[-1,1\right];Gabor_{odd,y}\approx G_{odd,y}=\left[\begin{array}{c} -1\\ \;1 \end{array}\right] \end{array}$$

(16) $$\begin{array}{l} Gabor_{even,x}\approx G_{oeven,x}=\left[-1,\;2,-1\right];Gabor_{even,y}\approx G_{even,y}=\left[\begin{array}{c} -1\\ 2\\ -1 \end{array}\right] \end{array}$$

The above discretization of the Gabor filters: *G*_*odd,x*_ and *G*_*odd,y*_ also represent the discrete gradient operator ∇ :

(17) $$\begin{array}{l} \nabla I=\left(\nabla_{x}I,\nabla_{y}I\right)=\left(I*G_{odd,x},I*G_{odd,y}\right) \end{array}$$

The structure of the oriented-double-opponent receptive field can be seen as a filter which acts as a second derivative in both the spatial and chromatic domains.

### Weights of Modified Edges

In order to calculate the response of an opponent channel to a Gabor RF on different scales, Equation (5), we use a Gaussian Pyramid (Adelson et al., 1984). In this way, the image is down-sampled instead of up-sampling the Gabor filter.

(18) $$\begin{array}{l} R_{c,i}=|GaussianPyramid{\left\{OP_{c}\right\}}_{\sigma_{i}}*Gabor_{even}\left(\theta \right)| \end{array}$$

### Filling-In Process

The divergence operator, div Equation (10), is computed as:

(19) $$\begin{array}{l} div\left(F\right)=\frac{\partial F}{\partial x}+\frac{\partial F}{\partial y}=F*G_{odd,x}+F*G_{odd,y} \end{array}$$

Where F is an image input.

Therefore, Equation (10) can be written as:

(20) $$\begin{array}{l} △I_{op}=\nabla^{2}I_{op}=\text{div}\left(\text{Trig}\right)=Trig_{x}*G_{odd,x}+Trig_{y}*G_{odd,y} \end{array}$$

### Parameters

We performed a set of simulations in order to determine the constants α and β. We found that increasing the β parameter (increasing the weight of the modified gradient, *ODO*, Equation 8) increases the saturation of the predicted result (since the level of the relevant gradient is increased). This means that choosing a higher value for β increases the saturation of the filled-in predicted color and also increases its intensity while preserving its hue. The α parameter affects the magnitude of the original gradient of the original stimulus. We arrived at the conclusion that the ratio between α and β determines the level of the filled-in predicted saturation. In all the simulations presented here α = 1 and β = 0.5.

### Comparison to Psychophysical Findings

In order to compare the predictions of the model to psychophysical findings we created sets of images that contain the same color values that have been used in previous psychophysical experiments (Devinck et al., 2005; Kimura and Kuroki, 2014b). Each color value used in the stimulus was converted from the CIE Lu'v' 1976 color space to the sRGB color space, in order to create the input images for the model. The model was then applied to each image stimulus, and the predicted colors were calculated and converted back to the CIE Lu'v' 1976 color space. These CIE Lu'v' 1976 color values are presented in the results section.

## Results

The results present the simulations of the model through its equations (according to the Methods section) implemented by MATLAB software. The model's equations were solved in a similar way to that reported in “Methods for Solving Equations” (Simchony et al., 1990) but another option was through “Poisson Image Editing” (Pérez et al., 2003).

### Model's Simulation and Predictions

The model and simulation results (Figure 1G) are divided into three parts. The first part presents the model predictions for the assimilative (classic) watercolor effect. The second part presents the predictions of the model for the non-assimilative (non-classic) watercolor effect, while the third part presents the model predictions that relate to additional properties of the watercolor effect: the influence of the background luminance, and the effect of the inner color luminance on the perceived hue and the perceived brightness (Devinck et al., 2005, 2006; Cao et al., 2011; Kimura and Kuroki, 2014a,b).

#### Predictions—Assimilative (Classic) Watercolor Effect

**Figure 3:**
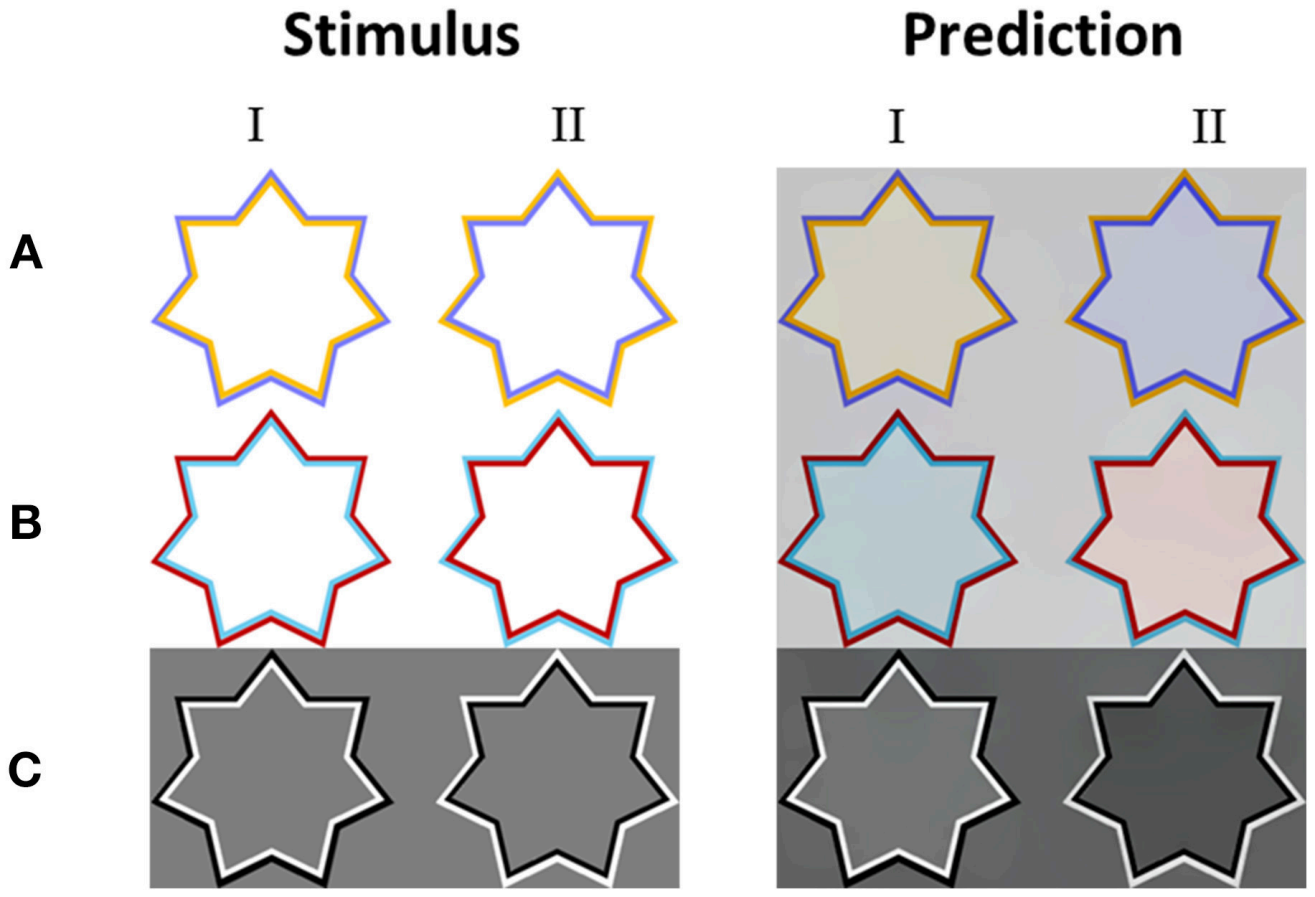
The model's predictions for assimilative watercolor stimuli. **(A)** The classic watercolor stimulus (left) and the model's predictions (right). **(B)** Additional example of an assimilative watercolor stimulus (left), with different colors, and the model's predictions (right). **(C)** An example of achromatic watercolor stimulus (left) and the model's predictions (right). Our model predicts that in the assimilative watercolor stimuli, the inner contour color is spread to the inner area of the stars.

The model simulations were tested on a large number of classic stimuli with a variety of chromatic thin polygonal curves (e.g., star shapes) that produce the watercolor effect. Figure 3 shows that the model succeeded in predicting the correct coloration of the classic assimilative watercolor effect. Note that the most of the assimilative watercolor effects present the complementary colors of the IC and the OC (the IC and the OC color are complementary in these stimuli). Our model indeed predicts a strong filling-in color response to such stimuli, Figures 3A–C.

Figure 3 demonstrates that the filling-in perceived color is more prominent in the predicted result (right side), which represents the model prediction for the corresponding stimulus, i.e., the original stimuli (left side). The filling-in effect of the stimuli with orange and purple polygonal edges were obtained as expected, Figure 3A, as well as a reddish color and cyan, Figure 3B. The level of saturation in the simulation results can be controlled by the parameters α and β, Equation (8). We also tested our model with achromatic watercolor stimulus. Figure 3C shows that the model correctly predicts a perceived darker or lighter inner area, according to the luminance of the inner contour.

##### Comparison to psychophysical findings

**Figure 4:**
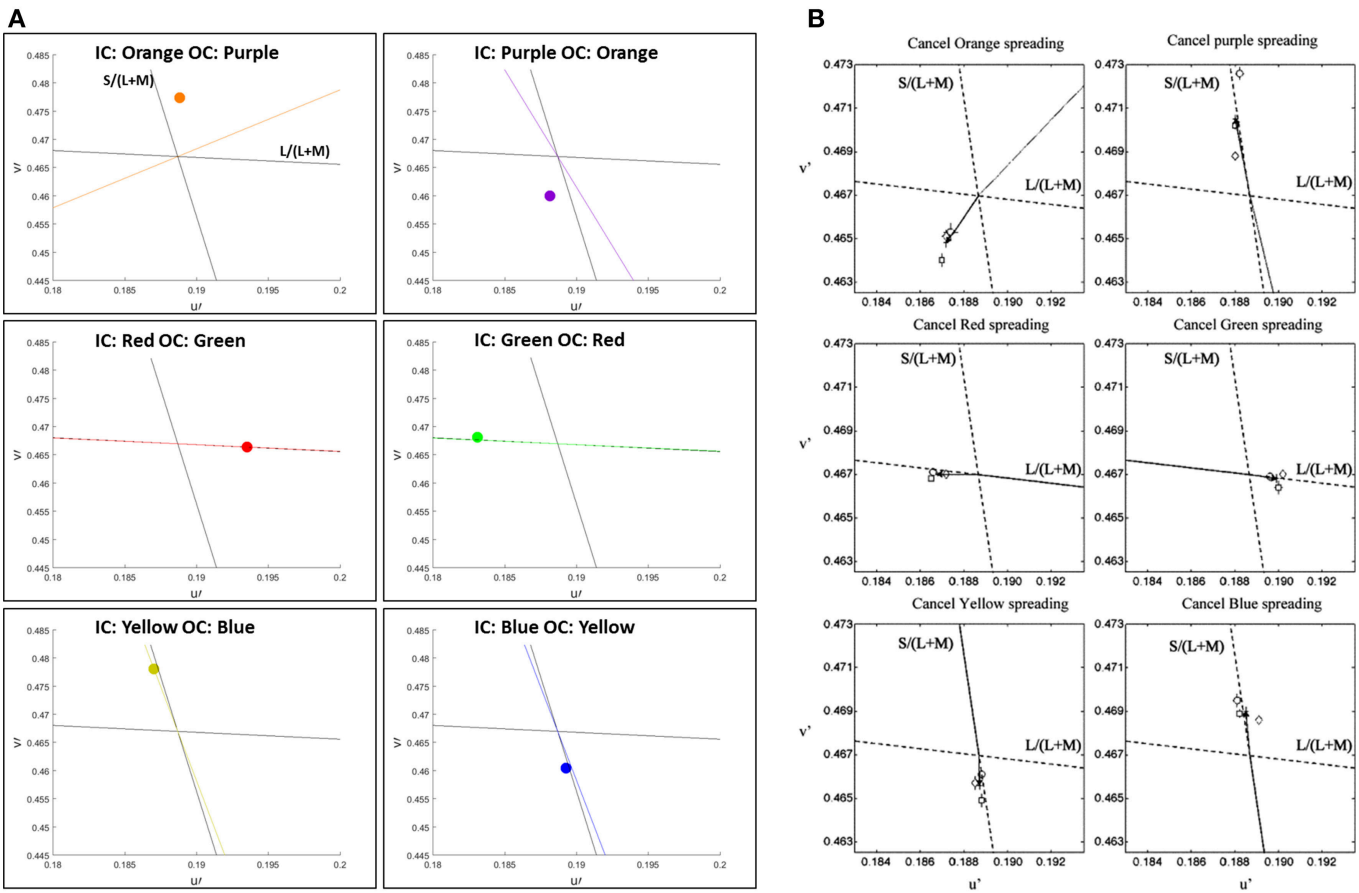
Comparison between the predictions of the model and the psychophysical findings of the assimilative effect, both presented in u'v' (CIELu'v' 1976) color space. The prediction of the model **(A)** and the chromatic cancelation data **(B)** that are taken from Devinck et al. (2005). Each row **(A,B)** presents a pair of IC and OC colors, which are orange–purple, red–green, and blue–yellow, respectively. The colored dots **(A)** represent the predicted results. The colored lines **(A)** represent the hue line of the IC contour color that was used in each pair of contours.

We confronted our model predictions with quantitative psychophysical results (Devinck et al., 2005). Figure 4 presents the predictions of the model in CIE Lu'v' (1976) coordinates instead of RGB images, see Methods. In order to enable the comparison between the model predictions and the psychophysical results, we applied the same set of colors as described in Devinck et al. (2005), as parameters to our model, see Methods.

Figure 4 demonstrates the comparison of the model prediction with Devinck et al. (2005) findings, which tested the assimilative effect on three pairs of colors: Orange and Purple, Red and Green, and Blue and Yellow. Note that, the psychophysical findings are obtained from a hue cancellation test and therefore represent the complementary colors of the perceived colors; however, our results represent the predicted perceived colors. Most of the predicted colors, Figure 4A, are in agreement with the psychophysical findings, Figure 4B. Only in the orange and the purple stimuli pair the predicted color is slightly more yellowish then in the psychophysical findings for the IC: Orange OC: Purple stimulus (Figure 4A top left) and slightly more bluish then in the psychophysical findings for the IC: Purple OC: Orange stimulus (Figure 4A top right).

#### Predictions—Non-assimilative (Non-classic) Watercolor Effect

**Figure 5:**
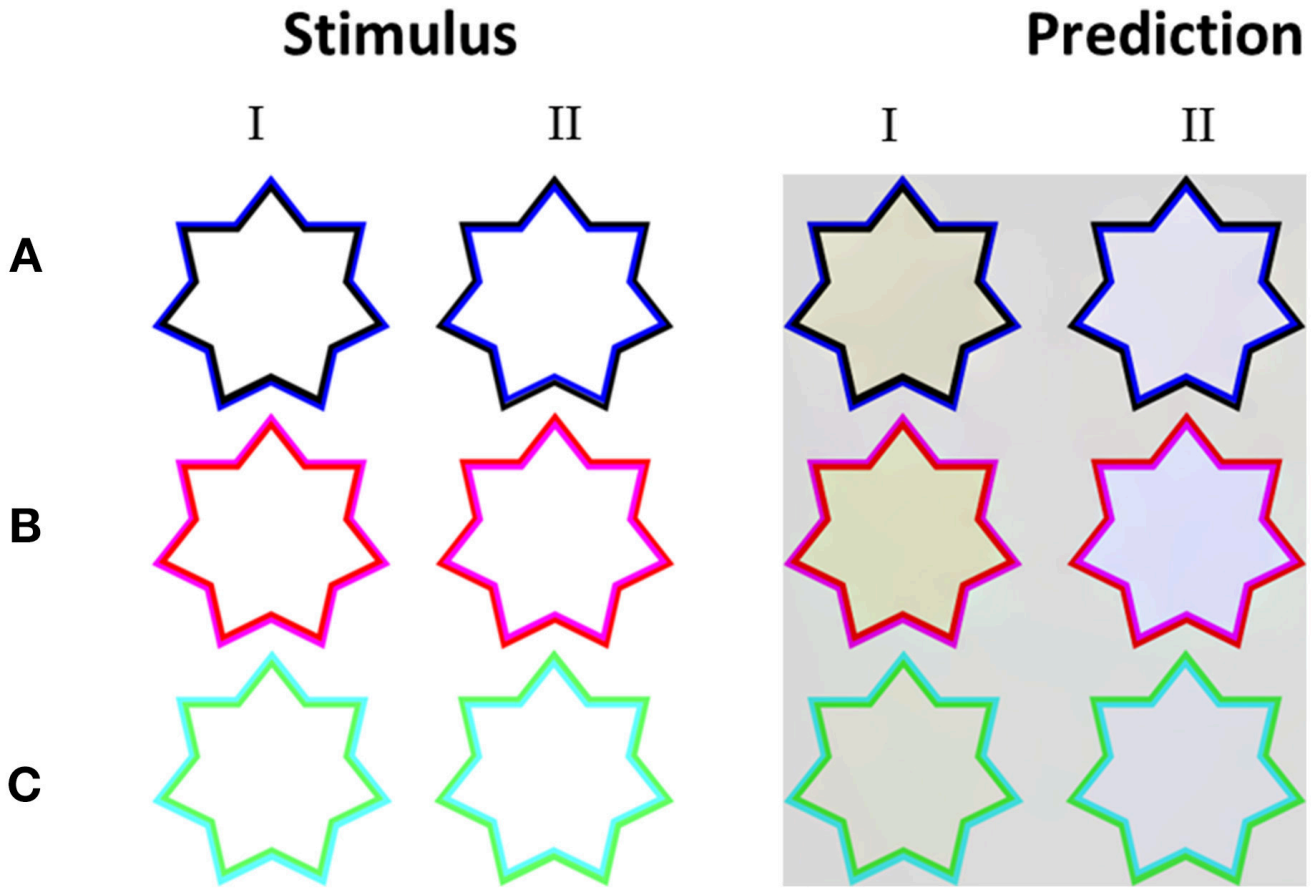
The model's predictions for non-assimilative watercolor stimuli. Each row presents different color variation of the inner and outer contours **(A–C)**. Column I presents color configurations which produce a perceived non-assimilative effect, while column II presents color configurations that produce a perceived assimilative effect, even though the diffused perceived color does not reflect the color of the inner contour only. The colors predicted by the model are yellowish in the non-assimilative configurations (I), and blueish in the opposite assimilative configuration (II).

We also tested two known versions of the non-assimilative watercolor effect (Pinna, 2006; Kimura and Kuroki, 2014a). In this case, we chose to test the three chromatic stimuli colors as tested originally by Kimura and Kuroki (2014a) for the non-assimilative watercolor effect. The stimuli in these versions have chromatic and achromatic edges/contours (Figure 5A) or specific pairs of colors (Figures 5B,C).

Kimura and Kuroki (2014a,b) psychophysically tested stimuli similar to those in Figures 5A,**B** and found that the induced colors were yellowish. The psychophysical results also demonstrated that a stimulus such as that in Figure 5A (left star), yielded a complementary color (yellowish) to the OC (bluish). Our model correctly predicts these complementary perceived coloration effects (filling-in effect), Figure 5A (left star).

Again, in accordance with psychophysical findings, our model could also correctly predict the influence exerted by the location of the chromatic contours, as to whether the same or complementary filling-in color is perceived in the inner area (Pinna, 2006; Kimura and Kuroki, 2014a), Figure 5A.

Kimura and Kuroki (2014a) observed that the perceived colors were not necessarily the “same” as or “complementary” to the IC/OC, but could be a combination of the IC and OC colors, Figures 5B,C (left stars). In agreement, the model results (Figure 5II) show indeed that the perceived color is determined by combination of the outer and the inner contours. In Figure 5B (left star), for example, the red IC contributes the same (red) color to the coloration effect, while the magenta OC contributes its complementary color (green). An additive combination of red and green colors yields a perceived yellowish coloration (Berns, 2000). These results are consistent with the model principles and Equations [Filling-in process; Equation (10)], such that both the IC and OC contours contribute as triggers to the filling-in process. The model correctly predicts the general trend that has been shown in previously reported experimental results (Pinna and Reeves, 2006) where the perceived chromatic filling-in color was determined by the combined influence of the chromatic and achromatic edges.

##### Comparison to psychophysical findings

Furthermore, we confronted our model predictions with quantitative psychophysical results (Kimura and Kuroki, 2014b). In order to enable the comparison between the model predictions and the non-assimilative watercolor effect experiment results, we applied the same set of colors as described in the results of Kimura and Kuroki (2014b), as parameters to our model, see Methods.

**Figure 6:**
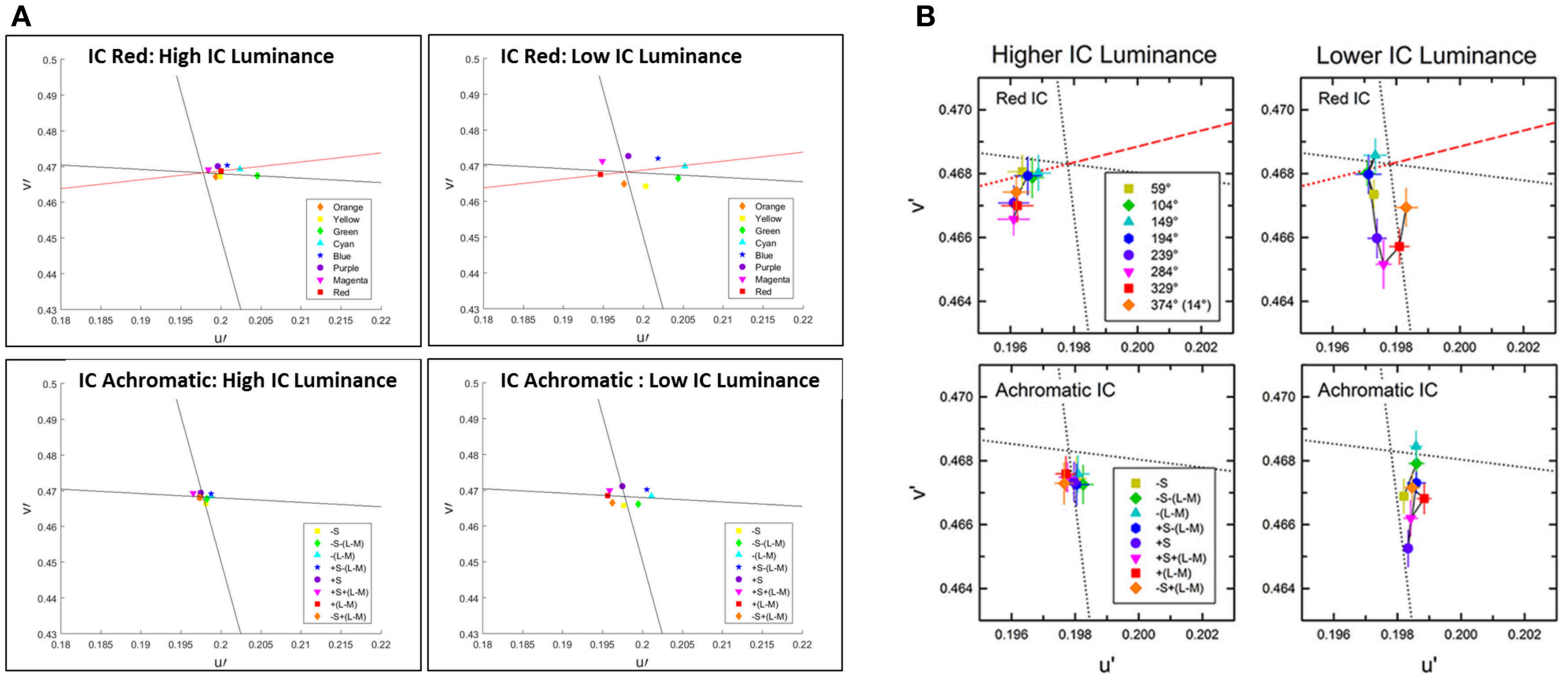
Comparison between the predictions of the model and the psychophysical findings for the assimilative and non-assimilative effects. The prediction of the model **(A)** and the chromatic cancelation data **(B)** where done for 8 different colors of the OC, similarly as Figure 4 Kimura and Kuroki (2014b). Top row **(A,B)** presents the experimental **(B)** and the predicted **(A)** results to stimuli with red IC. Bottom row present the experimental **(B)** and the predicted **(A)** results to stimuli with achromatic IC and the 8 different colors for the OC. In the left Column at each subfigure **(A,B)** the luminance of the IC is higher than the luminance of the OC. In the right column at each subfigure **(A,B)** the luminance of the IC is lower than the luminance of the OC as in Kimura and Kuroki (2014b).

The psychophysical experiments of Kimura and Kuroki (2014b) investigate both the assimilative and the non-assimilative effects as well as the role of intensity in the perceived effect. Figure 6 presents the model predictions and the results of Kimura and Kuroki (2014b) on a large repertoire of stimuli.

Figure 6 presents the predicted (A) and experimental results (B) of stimuli that share the same IC color at each sub-figure while the experiment tested 8 different OC colors. The top row presents the results for the red IC color and the bottom row presents the result for the achromatic IC color, while the outer color was presented with different chromatic colors. Left column presents the result when the IC color has a higher luminance level and the right column present the results when the IC color has a lower luminance level.

The stimuli with higher luminance of the red IC (Figure 6B) yielded perceived colors which were ranged from red to orange. Therefore, this trend of results shows an assimilative reddish color effect. The predicted result (Figure 6A) shows assimilate effects in adjustment to the red line. However, the perceived color is more reddish than orange as in the experimental results (Figure 6B). The stimuli with lower luminance of the red IC (Figure 6B) yielded an oval shape adjacent to the -S line. Our result also predicts an oval shape, but the shape is adjacent to the L line. It will be discussed in Discussion. The stimuli with higher luminance of the achromatic IC yielded a small magnitude of the perceived effects, in both the experimental (Figure 6B) and the predicted (Figure 6A) results. However, in the experimental results the effects slightly tend to be yellowish, while in the predicted results the effect is almost invisible (no filling-in effect). The stimuli with lower luminance of the achromatic IC also yielded a yellowish perceived color in the experimental results. In the predicted result the predicted colors are the complementary colors of the OC. It has to be noted that the achromatic configuration of the experimental result were tested also in additional studies such as Pinna (2006) and Hazenberg and van Lier (2013), and their trend of results are in better agreement with the prediction of the model (Figure 6A), see Discussion.

#### The Role of the Luminance Contrast Between the IC and the OC

**Figure 7:**
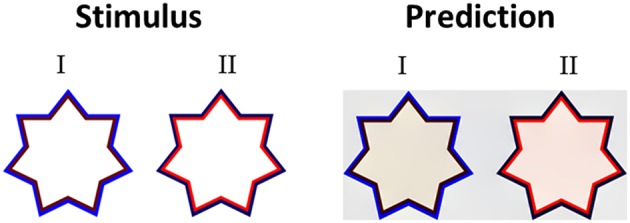
The role of the luminance level of the IC and the OC. In both stimuli (I,II) the hue of the IC is reddish and the hue of the OC is bluish, but with a different level of luminance intensity. In stimulus I, the IC has a low luminance level (dark red), while the OC has a high level of intensity. The predicted color is yellowish (I right), thus the perceived effect is a non-assimilative effect. In stimulus II, the IC has a high luminance level, while the OC has a low intensity (dark blue). The predicted color is reddish (II right), thus the perceived result is due to an assimilative effect.

Having discussed the model's predictions to highly saturated stimuli from the literature with different variations of chromatic properties (Figures 3, 5) we then tested the model's predictions for stimuli with different luminance as well as different chromatic properties. Devinck et al. (2005) and Pinna et al. (2001) showed that the magnitude of the filling-in effect increases with increasing luminance contrast between the relevant contours. Our model predicts this effect of luminance contrast between the IC and OC. Figure 7 presents the model predictions to a “switching” effect (non-assimilative: Figure 7I vs. assimilative: Figure 7II) whereby the luminance contrast determines whether the perceived effect will be assimilative or non- assimilative (Kimura and Kuroki, 2014a). Even though the IC color in both stars is reddish and the OC color blueish, the predicted colors are different (pale yellowish in the left star and pale reddish in the right star), Figure 7. It should be noted that in this case, the model's prediction is in agreement with the experimental results of Kimura and Kuroki (2014a) that showed that the luminance condition suitable for the non-assimilative color spreading is the reverse (in their Weber contrast) of the assimilative color spreading. We argue that these experimental findings (Kimura and Kuroki, 2014a) shed a new light on the common assumption in the literature that assimilative and non-assimilative are different effects and are derived from different mechanisms (Kimura and Kuroki, 2014a,b). This topic will be discussed in more detail in the Discussion.

An additional important finding relates to the claim that only the assimilative type of watercolor effect is possible when the IC and the OC have the same level of luminance (Devinck et al., 2005). Accordingly, our model predicts that the assimilative effect should be perceived under such iso-luminance conditions and also predicts that the effect will be weaker than when the IC and the OC have different luminance values.

#### The Role of the Luminance Contrast Between the Background and the Contour

Several experimental studies that tested the role of background luminance on the perceived watercolor effect (Devinck et al., 2005; Cao et al., 2011; Kimura and Kuroki, 2014a) reported that the luminance contrast between the IC and the background, and between the OC and the background have a significant influence on the perceived effect.

**Figure 8:**
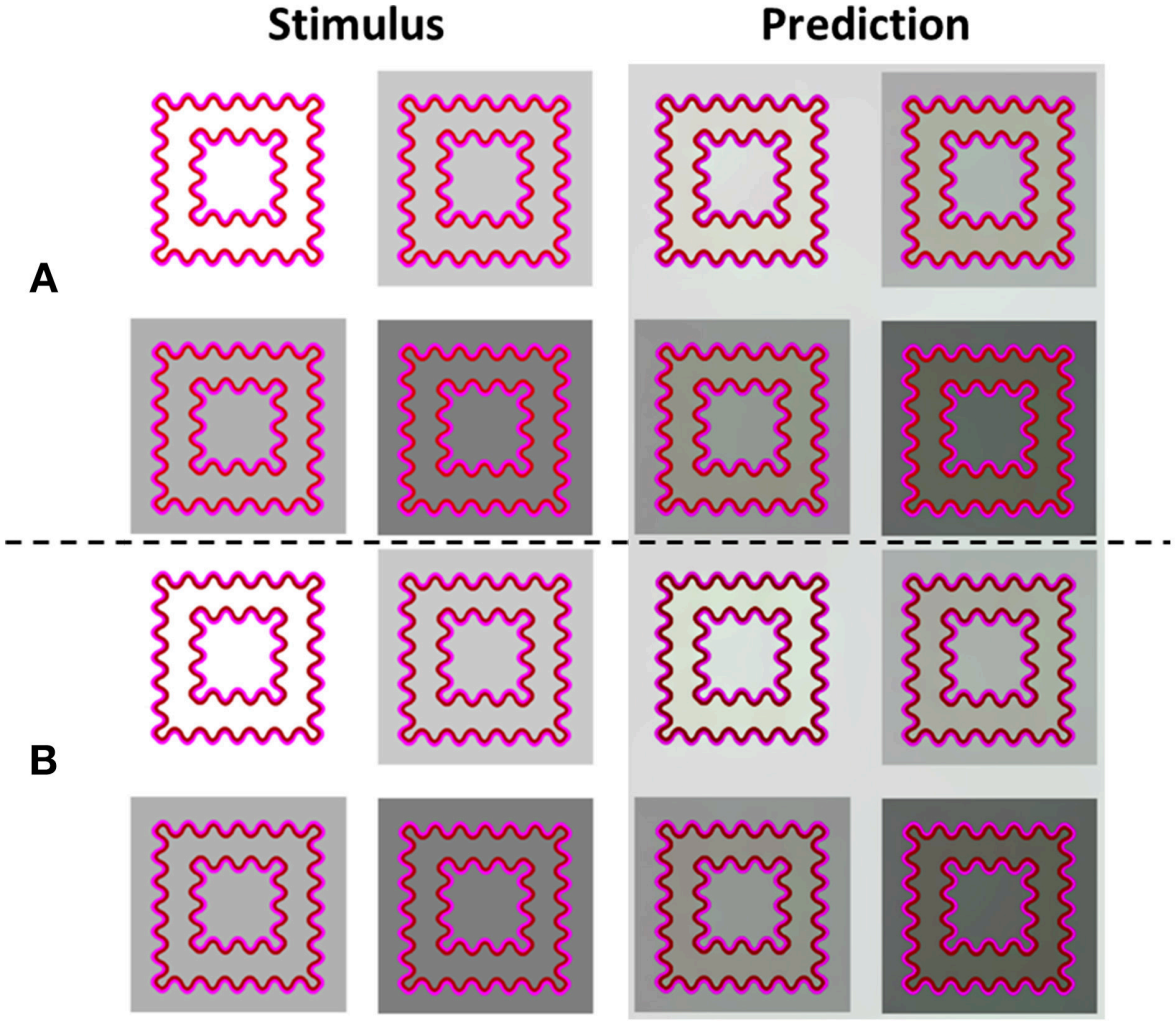
The influences of the background luminance and the luminance ratio between the IC and the OC on the predicted filling-in colors. The left column (Stimulus) presents the original stimuli. The right column (Prediction) presents the model's predictions. The IC/OC ratio is higher in rows **(A)** than in rows **(B)**, because the IC is darker in **(B)** than in **(A)**. When the IC is darker **(B)**, the predicted color is greenish (more prominent in the predicted images, Prediction), while when the IC is lighter **(A)** the predicted color is yellowish, when the background luminance is high (**A**: upper left, Prediction) and greenish when the background is darker.

Figure 8A presents the model's predictions for a response to the same stimuli used by Kimura and Kuroki (2014a), indicating that when the background is white (high luminance), the perceived color is yellowish. In contrast, when the background is darker (low luminance, Figures 8A,B), there is a tendency to a more greenish perceived color. This is because a change in the luminance of the background produces a change in the contrast between the contours (IC and OC) and the background, which in turn, influences the perceived effect. Importantly, the changes in perceived color predicted by the model were in accordance with the experimental results (Kimura and Kuroki, 2014a).

Figure 8B demonstrates that there are three options for luminance contrast that play a role in the watercolor effect. The first one is the contrast between the IC and the OC, the second, the contrast between the IC and the background, and the third one is the contrast between the OC and the background. In Figure 8B the luminance of the IC is lower than in Figure 8A. As a result, the perceived filling-in color appears greenish in the stimulus with the white background (high background luminance). In contrast, the perceived filling-in color in Figure 8A appears yellowish. These perceived coloration effects were intensified in the model's simulation (Figure 6 right) and support the suggestion that both the background and the luminance ratio between the IC and the OC contribute to the perceived effect. These predictions are in agreement with the psychophysical findings of Kimura and Kuroki (2014a).

## Discussion

We present here a generic computational model that describes the mechanisms of the visual system that activate the creation of chromatic surfaces from chromatic and achromatic edges. Our hypothesis was that these mechanisms can be revealed through a study of visual phenomena and illusions, such as the assimilative and non-assimilative watercolor effect and the Craik–O'Brien–Cornsweet (COC) illusions. The suggested model can be divided into two stages (or components). The first component determines the dominancy of the edges that trigger a diffusive filling-in process. The second component performs the diffusive filling-in process, which triggers the diffusion by heat sources. This process is modeled by the Poisson equation. The diffusion process is actually the same mechanism described for the afterimage effect (Cohen-Duwek and Spitzer, 2018).

In order to test the hypothesis, we developed a computational model that is able to predict both the assimilative and the non-assimilative watercolor effects. The model predictions, which are supported by psychophysical experiments (Pinna et al., 2001; Devinck et al., 2005, 2006; Pinna and Grossberg, 2005; Pinna and Reeves, 2006; Cao et al., 2011; Coia and Crognale, 2014; Kimura and Kuroki, 2014a,b), argue that both the assimilative and non-assimilative watercolor effects are derived from the same visual mechanism. In addition, the model can successfully predict quantitatively and qualitatively the psychophysical results reported by many researchers, such as the influence of the background luminance, contour intensities, contour saturations, and the relationship between them (Pinna et al., 2001; Devinck et al., 2005, 2006; Pinna and Grossberg, 2005; Pinna and Reeves, 2006; Cao et al., 2011; Coia and Crognale, 2014; Kimura and Kuroki, 2014a,b).

### Comparison to Other Models

The only computational model in the literature, that is relevant to the watercolor effects, is the FACADE model (Pinna and Grossberg, 2005). In a more recent publication of Pinna and Grossberg (2005), the FACADE model was challenged by testing several stimulus parameters acting in the watercolor effect, such as the role of the contrast between the IC and the OC, the role of the background luminance, and different shape variations of the stimulus. While the FACADE model could predict the results of the stimuli on the assimilative watercolor effect it was not designed to, and indeed was unable to, predict the non-assimilative watercolor effect and its properties.

The FACADE model comprises two components. The first component, the BCS, detects the borders that block the diffusion process. The second component, the FCS, spreads the color to all directions until it is blocked by edges. The FACADE model is unable to predict the non-assimilative effect first because the spread of color is derived from the chromatic surface itself, and there is no mechanism that creates complementary colors. A second reason is that the border, which is detected by the BCS, prevents the OC color of the watercolor effect from spreading inside the inner area of the stimulus.

The ability of the FACADE model to predict only the assimilative effects (Pinna and Grossberg, 2005; Pinna, 2006; Cao et al., 2011; Kimura and Kuroki, 2014a,b) has contributed significantly to the general consensus in the literature that the assimilative and non-assimilative effects are derived from different mechanisms. In contrast, Kimura and Kuroki (2014a) found strong psychophysical evidence that assimilative and non-assimilative effects both share the same Weber contrast rule under specific psychophysical constraints. However, despite these Weber rules, they concluded that the effects might still involve different mechanisms.

Unlike FACADE, two factors allow our model to predict the non-assimilative watercolor effect. First, each edge in the stimulus triggers a diffusion process. Therefore, each edge contributes to the achromatic areas i.e., the inner area and the outer area. The color adjacent to the achromatic area contributes its color i.e., triggers a diffusion process of the same color, to this area; while the color in the other side of the edge contributes the complementary color to the same area. In other words, the color in the outer side of an edge triggers a diffusion process of its complementary color. The reason why the complementary color is obtained from the model is explained in the Model section. The exact colors that will be spread are calculated by the responses of the double-opponent RFs, Equations (8–10). The resultant colors, are therefore not necessarily exactly the “same” or “complementary” to the IC/OC, but rather a linear combination of the colors of the IC and the OC. In addition, the model assumes that the main role of the contours is to trigger the diffusion process as “heat sources,” (Equation 10), and not as primarily designed to block the diffusion process.

It could be claimed that additional computational models that have been suggested for edge integration should be regarded here as competitors, which can explain this filling-in mechanism of chromatic and achromatic surfaces. Rudd (2014) summarized and discussed several computational models designed to perform the edge integration function in the visual system. He argued against the idea that the filling-in effect results from the activation of a low visual spatial frequency channel, due to the fact that the spatial extent of the filling-in effect is far larger than the area or distance spanned by the lowest spatial frequency filters in human vision (about 0.5 cycle/degree) (Wilson and Gelb, 1984). It should be noted that the watercolor effect has been shown to spread over 45° (Pinna et al., 2001), a spatial range that is not consistent with a low spatial frequency of the visual system.

**Figure 9:**
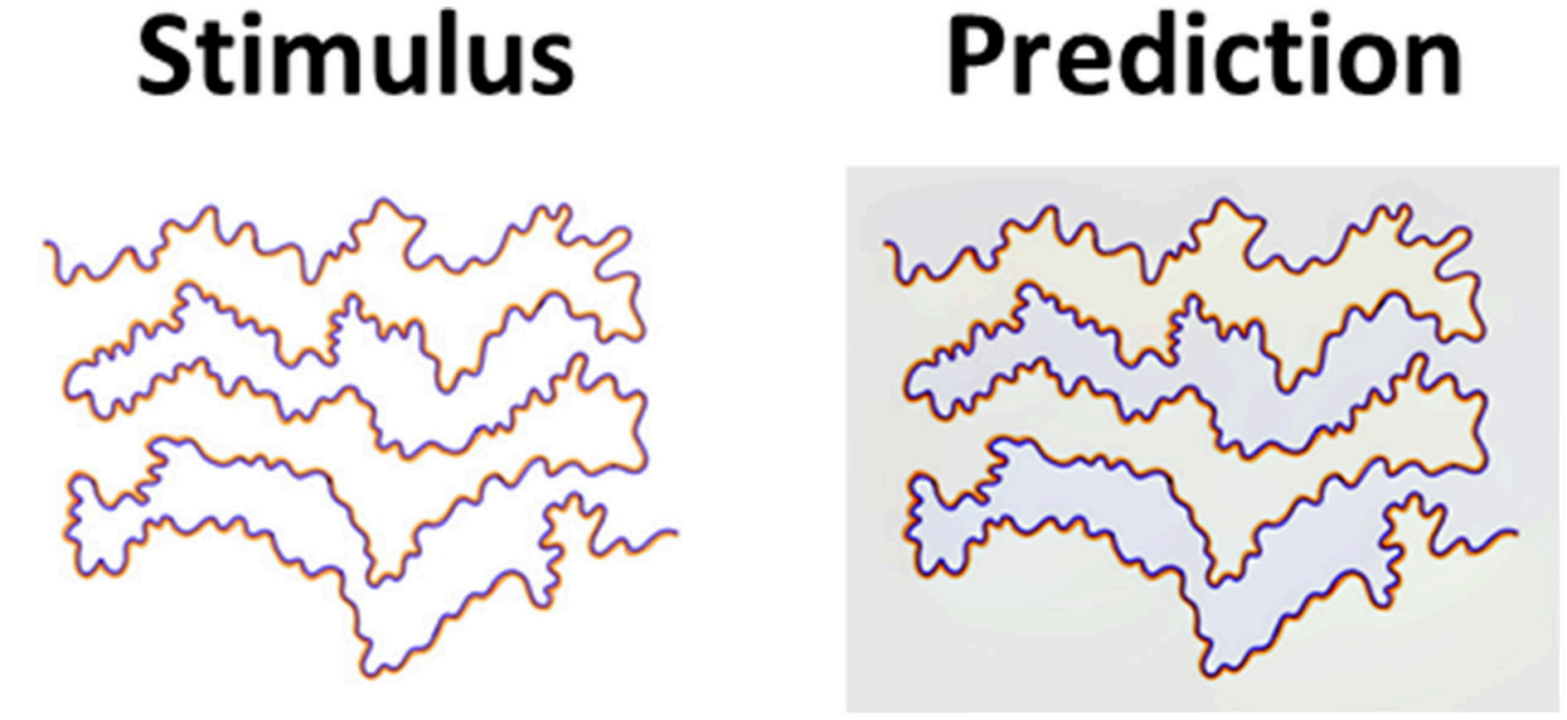
The watercolor effect with open boundaries. The left column (Stimulus) presents the original watercolor stimulus with open boundaries (Pinna et al., 2001). The second column (Prediction) presents the model's prediction. Even with open boundaries, the filling-in is perceived (Stimulus), as correctly predicted by the model (Prediction).

Although Rudd (2014) also argued against the diffusive filling-in mechanism, we believe that his justification was based on the specific diffusive FACADE model suggested by Grossberg and his colleagues (Grossberg and Mingolla, 1987; Grossberg, 1997; Pinna and Grossberg, 2005). According to FACADE, the chromatic edges function as borders to block the diffusive process. If the watercolor stimulus is open (unclosed boundaries), the FACADE model predicts that the color would leak from the open ends, which, in reality, does not occur. In contrast, our diffusive computational model does not fail in such a case. Figure 9 demonstrates that our model successfully predicts this effect, because the edges in our model are used as triggers, Equation (10), rather than borders for diffusion.

Rudd (2014) suggested a qualitative “Edge integration” model, through long range receptive fields in area V4 (Roe et al., 2012). Rudd suggested that lightness and darkness “edge integration” cells in V4 could integrate the responses of V1 simple receptive fields with a light or dark direction toward the center of the V4 receptive field. An additional neuron in the higher level of the visual pathway hierarchy then integrates these receptive fields, and performs a subtraction operation between the lightness and the darkness “edge integration” receptive fields. This model qualitatively predicts specific induction effects [Figures 2, 9 in Rudd (2014)] but fails to predict classic filling-in effects, such as the watercolor illusion that manifest filling-in in all directions and over very wide spatial regions.

Since Rudd (2014) related the induction effects to filling-in phenomena, he supplied an additional argument against the diffusive filling-in model, which is based on the model of Grossberg (Grossberg and Mingolla, 1987; Grossberg, 1997; Pinna and Grossberg, 2005). This argument is related to the FACADE model's failure to predict the specific induction effects, [Figure 2 in Rudd (2014)] and Figure 9.

**Figure 10:**
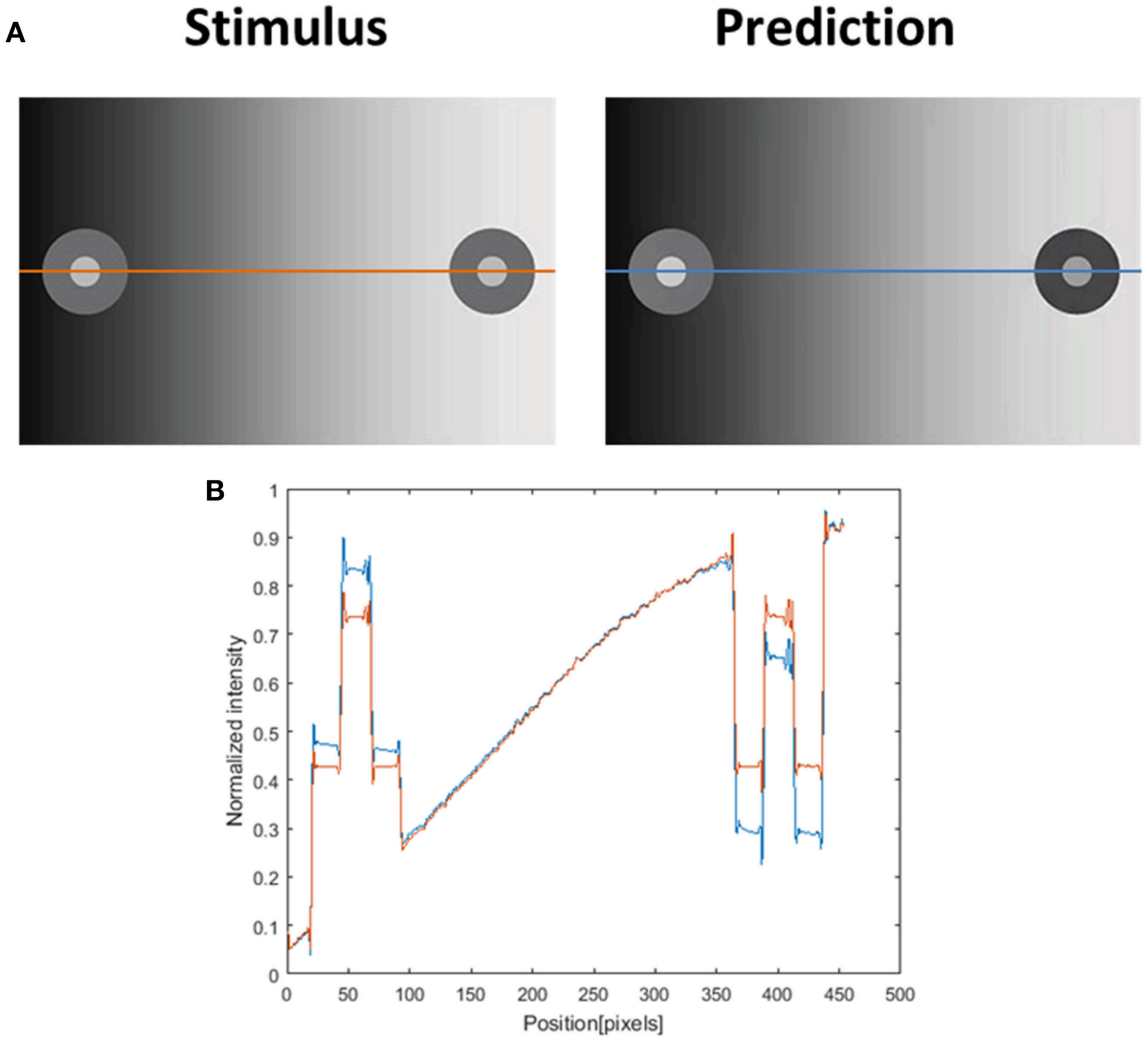
Induction effect and the model's prediction. **(A)** The original induction stimulus from Rudd (2014) (Stimulus), and the model's prediction (Prediction). The second row **(B)** presents the luminance of the original image (orange line) and the predicted perceived luminance (at blue line) along the orange and blue axes in **(A)**. The predicted perceived luminance [demonstrated along the blue line in **(A)**] is higher than the original luminance [demonstrated along the orange line in **(A)**] in the left disk (including the inner circle and the outer ring of the disk), and shows a lower level of luminance than the original value in the right disk.

There is currently a disagreement in the literature as to whether these specific induction effects are the result of a filling-in mechanism, an adaptation mechanism of the first order (Spitzer and Barkan, 2005), or a local or (remote) contrast mechanism (Blakeslee and McCourt, 1999, 2001, 2003, 2008). We argue that a visual effect may not necessarily be determined by a single dominant mechanism, and that several mechanisms could be involved. Different mechanisms could give rise to contradicting effects on one hand, or alternatively could work in synergy to enhance the perceived effect. An interesting question is whether this induction effect can also be predicted by our proposed model. Figure 10 demonstrates that our filling-in model can predict the first order variation of the specific induction effect, [Figure 2 in (Rudd, 2014)]. Since this effect is predicted by our filling-in model, and also by an adaptation of the first order model (Spitzer and Barkan, 2005), we believe that the induction effect can be attributed to both mechanisms.

Experimental results show that the size of the inducer areas and the size of the induced area play a crucial role in the perceived induction effect (Shevell and Wei, 1998). The suggested filling-in model is based on edges that trigger a diffusion process, therefore the size of the induced area and the size of the inducer area do not play a role in our filling-in model. However, these two spatial factors do play a major role in the adaptation of the first order mechanism (Spitzer and Barkan, 2005).

We believe that there is a certain confusion in the literature regarding the source and the mechanisms of the induction and the filling-in effects. Kingdom (2011), for example, argued in his review that: “…‘filling-in’ of uniform regions is mediated by neural spreading has been seriously challenged by two sets of findings: 1. That brightness induction is near-instantaneous and 2. That the Craik–Cornsweet–O'Brien illusion is dependent on the presence of residual low-frequency information and is not disrupted by the addition of luminance noise. ‘Filling-in’ should at best therefore be considered as a metaphor for the representation…”. We argue that these claims are problematic, based on different psychophysical results (Pinna et al., 2001), and also query the feasibility of a mechanism, which is based on spatial filtering.

Kingdom (2011) assumed that these two effects of induction and other filling-in effects (the COC effect) derive from the same mechanism. For this reason, he argued against a diffusive filling-in mechanism, since a diffusive process requires more time. Kingdom (2011) also based his arguments on the findings reported by Blakeslee and McCourt (2008) that the temporal response of the induction effect (simultaneous contrast) lagged by <1 ms. In contrast, Pinna et al. (2001) found that the temporal response of the watercolor effect is about 100 ms. We believe that there is no contradiction between the two temporal results (Pinna et al., 2001; Blakeslee and McCourt, 2008), since they are associated with two different mechanisms, namely induction and the diffusive filling-in process. The first mechanism (induction of the first order) (Spitzer and Semo, 2002; Spitzer and Barkan, 2005; Tsofe et al., 2009; Kingdom, 2011) occurs in/at early visual areas, such as the retina, while the second mechanism (COC or watercolor, diffusive filling-in) occurs in a higher visual area. In addition, the spatial filling-in spread of 45°, reported for the watercolor illusion cannot be explained by any receptive field or low-spatial frequency channel of the visual system (Rudd, 2014).

In this context, we contend that positive and negative aftereffects (such as in “color dove illusion” and the “stars” illusion) (van Lier et al., 2009; Barkan and Spitzer, 2017), are perceived as a result of a diffusive filling-in process that cannot be explained by any spatial filtering mechanism. The reasons for this are: (1) The perceived color is obtained in an area that has not been stimulated by any color, at the time that the color is perceived [aftereffect with filling-in as in the “color dove illusion” and Van Lier “stars” (van Lier et al., 2009; Barkan and Spitzer, 2017)]. (2) The location of the achromatic reminder contour determines and triggers the perceived color. The filling-in model proposed here shares the same diffusion component, Equation (10), as suggested for the positive and the negative aftereffects (Cohen-Duwek and Spitzer, 2018). Although Kingdom (2011) supported the description of the filling-in and induction events by the filter models of Blakeslee and McCourt (2008), their model cannot predict the assimilative and the non-assimilative watercolor effects, or the aftereffects.

### Predictions for Watercolor Properties

Having discussed the options of various alternative models for the “filling-in” phenomena, we were interested to test our model's predictions with studies that define general properties and rules for the watercolor effect, although without a computational model (Kimura and Kuroki, 2014a). We have already described the success of our model in correctly predicting experimental results (Kimura and Kuroki, 2014a) demonstrating crucial properties regarding the strength of the watercolor effect and its relation to the assimilative and non-assimilative effects. We explain below how the basic structure of the suggested model can explain these findings, without requiring any additional components.

***Complementary colors***: Several studies have demonstrated that a maximal filling-in response is perceived when the IC and the OC have complementary colors (Pinna et al., 2001; Devinck et al., 2006) and it should be noted that the model correctly predicts this trend, Figure 6. This can be explained by the model equations (Equations 3–10), through solving the Poisson equation. The IC triggers an assimilative filling-in (of the same color as the IC) toward the inner area, while the OC triggers a non-assimilative filling-in, with the opposite color to the IC contour (Figure 2, i.e., its complementary color), toward the inner area. According to the model, if the color of the OC is complementary to the color of the IC, the combination of colors that diffuse to the inner area will be the same as the color of the IC (assimilative color) and complementary to the color of the OC, which makes it the same color as the IC again. Consequently, the perceived color is enhanced.

***Luminance contrast:*** Several studies have reported that the magnitude of the filling-in effect increases with increasing luminance contrast between the IC and OC contours (Pinna et al., 2001; Devinck et al., 2005; Devinck and Knoblauch, 2012). This property of the luminance contrast is treated similarly to the chromatic channels. The weights of the modified gradients calculation, Equations (7–8), gives greater dominancy to the gradients between the IC and the OC. It is therefore not surprising that the model correctly predicts the importance of the luminance contrast, between the IC and the OC, in the watercolor effect.

***Saturation:*** Devinck et al. (2006) showed that increasing the saturation of the outer and inner contours increases the shift in chromaticity of the filling-in effect. This information is included in the model through the chromatic opponent channel, Equation (3). Higher color saturation is expressed as a higher response in the chromatic opponent channels. This property has been tested and the model predictions show good agreement with the results of experimental studies.

***Weber rule – IC contrast/OC contrast:*** Kimura and Kuroki (2014a) reported that the ratio between the IC luminance contrast and the OC luminance contrast determines the perceived filling-in effect, Figure 8. The IC contrast is the Weber contrast of the chromatic IC luminance and the background luminance, while the OC contrast is the Weber contrast of the chromatic OC luminance and the background luminance, Equation (21). Note that since the background is achromatic, this Weber contrast is related only to the luminance domain. Kimura and Kuroki (2014a) argued that if the IC contrast is smaller than the OC contrast, an assimilative effect is perceived, Equation (21). In contrast, if the IC contrast is larger than the OC contrast, a non-assimilative effect is perceived, Equation (21).

(21) $$\begin{array}{l} \frac{\left|L_{IC}-L_{Bkg}\right|}{L_{Bkg}}<\frac{\left|L_{OC}-L_{Bkg}\right|}{L_{Bkg}}\to assimilative\;effect\\ \frac{\left|L_{IC}-L_{Bkg}\right|}{L_{Bkg}}>\frac{\left|L_{OC}-L_{Bkg}\right|}{L_{Bkg}}\to non-assimilative\;effect \end{array}$$

Where *L*_*IC*_, *L*_*OC*_, and *L*_*Bkg*_ are the luminances of the IC, OC, and the background, respectively.

Our model was tested with a variety of stimuli with different luminance backgrounds, different chromatic contours (Figures 8A,B), and different Weber ratios. Figure 8 demonstrates the predictions of the Weber contrast rule with non-assimilative effect. Additional stimuli were tested, but showed a smaller perceived effect. Interestingly, the Weber contrast rule and the predictions of our model do not necessarily always yield the exact assimilative or non-assimilative colors, but rather a different color as found experimentally (Kimura and Kuroki, 2014a). For example, the stimuli in Figures 8A,B have the same colors (red and magenta), but because the IC in Figure 8A has a higher luminance than the IC in Figure 8B, this gives rise to a yellowish color in Figure 8A but a greenish color in Figure 8B. Note that despite the difference in luminance levels, both effects share the same trend of Weber contrast rule, and thus both appear as non-assimilative effects. The model's predictions are in agreement with the Weber contrast rules (Kimura and Kuroki, 2014a), Figure 8. This demonstrates that both the model and the Weber contrast rule can predict in which contrast configuration the perceived effect is assimilative or non-assimilative.

Let us explain how our model can predict this Weber contrast rule. If an IC has a high value of Weber contrast, the “heat source” located on the edge between the IC and the background has the highest value and the diffusion process from this edge has a strong influence on the perceived color. Accordingly, the color spreading from this “heat source” (the edge between the IC and the background) to the inner area has the same color as the color of the background (white Figure 8A), and the complementary color of the IC (cyan—the complementary color of the red IC), Figure 8A. The cyan color, which is a combination of green and blue, contributes to this bluish-greenish perceived effect (Figure 8B).

**Figure 11:**
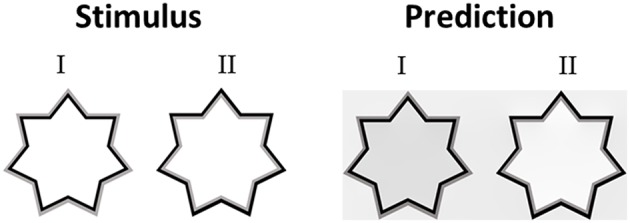
The model's predictions for chromatic watercolor stimuli. In the left column (Stimulus I), the luminance of the OC (gray) lies between that of the IC (black) and the background (white). On the right side, (Stimulus II) the luminance of the IC lies between that of the OC and the background. The predicted filling-in luminance inside the left stimulus (Prediction I) is lower than the predicted filling-in luminance inside the right stimulus (Prediction II). Note that the Weber contrast of the OC is smaller than the Weber contrast of the IC in the left stimulus (I), and larger than the Weber contrast of the IC in the right stimulus (II). The topic is discussed in the Discussion section of the text.

We were interested in whether the Weber contrast rule is applicable to the achromatic watercolor stimuli. Cao et al. (2011) conducted a psychophysical study in order to investigate the influence of the luminances of the IC, OC, and the background on the perceived achromatic watercolor effect. They found that the filling-in effect disappeared when the luminance of the OC was between the luminances of the IC and the background. Kimura and Kuroki (2014a) reported that the findings of Cao et al. (2011) are consistent with their psychophysical findings, and also with their suggestion for the role of the Weber contrast rule. The prediction of our model (Figure 11) is also in agreement with the Kimura and Kuroki (2014a) findings. In Figure 11, the luminance of the OC lies between that of the IC and the background. In terms of the Weber contrast rule, the Weber contrast of the OC is smaller than that of the IC. Therefore, such a configuration should lead to a non-assimilative perceived effect. However, since the perceived color inside the star is darker than the background (Figure 8I); this might be seen as a diffusive effect of the IC color (“assimilative” effect), which is black. According to our model, the perceived color is a combination of the same color as the IC (black) and the complementary color of the OC (gray, which is the complementary of gray), therefore, the model correctly predicts this effect. Accordingly, the terms “assimilative” and “non-assimilative” watercolor effects are not the precise terms regarding the perceived colors of the achromatic watercolor stimuli. It should be noted that there might be a dependency of the perceived effect on the stimulus size. This property should be further investigated experimentally.

Not all experimental studies agree about the perceived color in the non-assimilative watercolor effect (Pinna, 2006; Kimura and Kuroki, 2014b). Kimura and Kuroki (2014b), for example, claim that if the luminance of the IC is low (very dark IC), the perceived filling-in effect is predominantly yellow, regardless of the OC color. Kimura reported this finding to be inconsistent with previous results reported by Pinna (2006), which described a complementary color filling-in effect with black IC and chromatic OC combinations. Additional experimental study supports the results of Pinna (2006) and the idea that complementary colors are perceived, when the IC color is dark (Hazenberg and van Lier, 2013). The model results predict that the perceived colors are predominantly complementary to the OC colors, when the IC is dark. Even though the predicted results, Figure 6, are predominantly complementary to the OC colors, when the IC color is dark red, the predicted colors are slightly shifted to the red IC color. When the IC is achromatic the predicted colors, Figure 6, are the complementary colors to the OC colors.

Our model, thus, supports the findings of Pinna (2006) and Hazenberg and van Lier (2013), Figure 6, and is not in agreement with Kimura and Kuroki (2014b) because the chromatic OC triggers a filling-in effect that is complementary to the inner area, and therefore the perceived color will be complementary to the OC (the IC is achromatic and so does not contribute any color to the effect).

### Model's Predictions for the COC Effect

**Figure 12:**
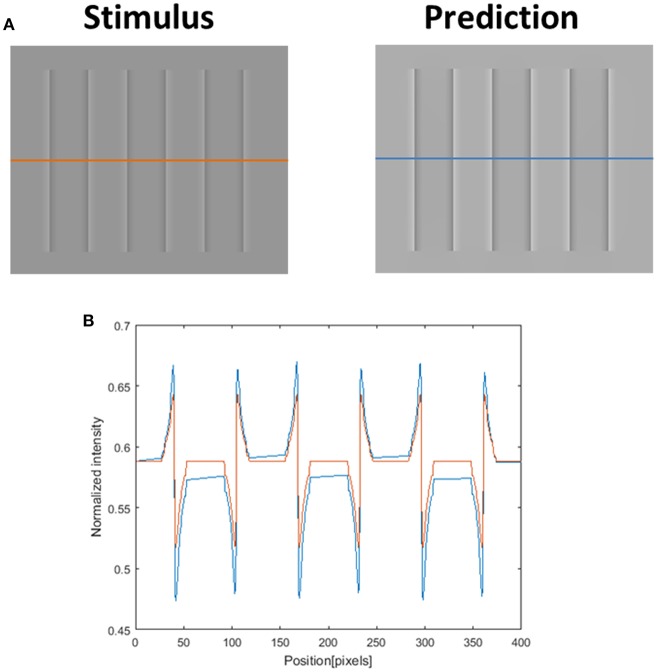
The model's ability to predict the Craik–O'Brien–Cornsweet (COC) effect. The left image **(A)** represents the original COC effect (Stimulus, left), and the model's prediction (Prediction, right). The lower row **(B)** presents the luminance of the original stimulus (orange line) and predicted perceived luminance (blue line) along the orange and blue axes in **(A)**.

Although our model is mainly concerned with the predictions of the watercolor illusions, there are a number of other examples of filling-in effects, including the COC effect. We believe that the COC effect is driven solely by a diffusion mechanism, since the physical stimulus in this effect is only an edge. The model prediction for the COC effect, which is demonstrated in Figure 12, uses the same set of parameters as the watercolor illusions (Figures 3, 5, 7–9, 11). Our suggestion that both phenomena (watercolor and COC) are related to the same visual mechanism, is in agreement with (Devinck et al., 2005; Todorovi, 2006; Cao et al., 2011) who showed that the watercolor stimulus profile is a discrete version of the COC stimulus profile. The success of the model prediction of the COC effect supports the suggestion that both effects (which are physically built only from edges) share the same “heat sources” diffusion mechanism, which is triggered by edges. The COC effect can actually be considered as a simpler case of the diffusive filling-in effect than the watercolor effects.

There are three main classes of computational models that have been used to investigate the COC effect. The first class is called the “Diffusive models” (Grossberg and Mingolla, 1987). Grossberg and Mingolla (1987) showed that the FACADE model can correctly predict the COC effect. Nevertheless, the FACADE model, in this case, can predict the COC effect when the stimulus contains open boundaries, but only through using an additional component that detects illusory contours, Figure 12. The illusory contours component will detect the illusory edges around the COC stimulus (Figure 12), and will prevent the color from spreading. However, this component is not necessary for the watercolor illusion, which can contain open boundaries. Figure 9 presents, for example, open boundaries, and it can be seen that there is no perceived effect of edge completion (illusory contour). It has to be noted that the suggested model does not include a component that detects illusory contours, and therefore our model does not predict filling-in effects that involve illusory contours e.g., “Neon Color Spreading.” Our model suggests that the illusory contours components are not necessary for the watercolor mechanism.

The second class of models is termed the “Spatial filtering models,” where these models utilize low-frequencies spatial filters in order to predict the filling-in effects (Morrone et al., 1986; Burr, 1987; Morrone and Burr, 1988; Ross et al., 1989; Blakeslee and McCourt, 1999, 2001, 2003, 2004, 2005; Dakin and Bex, 2003; Blakeslee et al., 2005; Kingdom, 2011). We argue that the spatial filtering approach has limitations in predicting the COC effect because the filling-in can be spread to sizes which cannot be explained by the sizes of the receptive fields that exist in the LGN or V1–V2 cortical areas. In addition, the COC effect can be obtained from edges that are built only from ODOG (Oriented Difference of Gaussian) filters (Blakeslee and McCourt, 1999).

The third class of models is termed the “Empirical models.” These models are designed to estimate the most likely reflectance values based on the pattern of the luminances observed in the image, together with learnt image statistics (Purves and Lotto, 2003; Brown and Friston, 2012). Typically, such an Empirical approach may explain why we perceive these visual effects, but cannot explain the neuronal mechanisms that lead to the perceived effects.

### Neuronal Sources of the Filling-In Effect

Studies designed to identify the neuronal source of the filling-in effects that are triggered by edges, e.g., the watercolor and the COC effects, can shed additional light on the possible neuronal mechanisms. A recent fMRI study (Hong and Tong, 2017) compared the responses of the visual areas (V1–V4) to real colored surfaces and to illusory filled-in surfaces, such as occur in the afterimage effect of van Lier “stars”(van Lier et al., 2009). Hong and Tong (2017) found a high correlation between the two types of stimuli, the real and the illusory, only in areas V3 and V4. They, therefore concluded that the perception of filled-in surface color occurs in the higher areas of the visual cortex.

Rudd (2014) suggested an “edge integration” model that works through long range receptive fields in area V4 (Roe et al., 2012). Both the qualitative (Rudd, 2014) model and (Hong and Tong, 2017) experiments support the idea that the source of the filling-in mechanism is located in V4. It has to be noted that our computational model can be regarded as this diffusion process but also does not contradict a mechanism of edge integration that can be derived from long range receptive fields (Rudd, 2014). This “edge integration” mechanism can also be symbolic and appear as a diffusion process.

As already discussed, we argue that both the watercolor effect and the COC effect share the same visual mechanism; therefore, we would expect to identify a similar neuronal source for both effects. A literature survey of experimental studies that investigated these sources revealed a lack of consensus regarding the neuronal source of the COC effect. A few studies reported that the effect occurs in low visual areas: the LGN, V1 and V2 (MacEvoy and Paradiso, 2001; Roe et al., 2005; Cornelissen et al., 2006; Huang and Paradiso, 2008), while other studies showed evidence that the effect occurs in higher areas of the visual system such as the V3 and caudal intraparietal sulcus (Perna et al., 2005). It is possible that there is no complete overlap between the cortical areas responsible for the COC effect and the watercolor effect, since the watercolor effect commonly involves color, while the COC effect involves achromatic stimuli.

Our model succeeds in predicting apparently conflicting perceived filling-in triggered-by-edges phenomena, e.g., the assimilative and the non-assimilative watercolor effects. The suggested mechanism is a filling-in process which is based on reconstruction of an image from its modified edges. The diffusion process, thus, is calculated by solving the heat equation with heat sources (Poisson equation). The edge of the trigger stimulus are modified by the model according to rules of dominancy, and computed as the heat sources in the Poisson equation. We therefore suggest that this model can predict all the filling-in-triggered-by-edges effect in both the spatial and temporal domains (Cohen-Duwek and Spitzer, 2018).

The challenge of “The interaction of the mechanisms underlying boundary and surface perception is an essential problem for vision scientists” has been presented previously (Cao et al., 2011). Here we introduce a new computational model that describes and predicts how any boundary can “create” surfaces by a filling-in process.

## Data Availability

All datasets generated for this study are included in the manuscript and/or the supplementary files.

## Author Contributions

All authors listed have made a substantial, direct and intellectual contribution to the work, and approved it for publication.

### Conflict of Interest Statement

The authors declare that the research was conducted in the absence of any commercial or financial relationships that could be construed as a potential conflict of interest.
